# Modifiable risk factors for diphtheria: A systematic review and meta-analysis

**DOI:** 10.1016/j.gloepi.2023.100100

**Published:** 2023-02-21

**Authors:** Juniorcaius Ikejezie, Busola Adebusoye, Winifred Ekezie, Tessa Langley, Sarah Lewis, Revati Phalkey

**Affiliations:** a Division of Epidemiology and Public Health, School of Medicine, University of Nottingham, UK; b Climate Change and Health Unit, UK Health Security Agency, London, United Kingdom; c Heidelberg Institute of Global Health, University of Heidelberg, Germany

**Keywords:** Diphtheria, Risk factors, Infectious disease, Systematic review, meta-analysis

## Abstract

**Objective:**

To identify modifiable risk factors for diphtheria and assess their strengths of association with the disease.

**Methods:**

This review was conducted in accordance with the Preferred Reporting Items for Systematic Reviews and Meta-Analysis (PRISMA) statement. Electronic databases and grey literature were searched from inception until January 2023. Studies had to report on diphtheria cases and estimates of association for at least one potential risk factor or sufficient data to calculate these. The quality of non-ecological studies was assessed using the Newcastle-Ottawa Scale (NOS), while the quality of evidence was evaluated using the Grading of Recommendations Assessment, Development, and Evaluation (GRADE) criteria.

**Results:**

The search yielded 37,705 papers, of which 29 were ultimately included. All the non-ecological studies were of moderate to high quality. Meta-analysis of 20 studies identified three factors increasing the risk of diphtheria: incomplete vaccination (<3 doses) (pooled odds ratio (POR) = 2.2, 95% confidence interval (CI) = 1.4–3.4); contact with a person with skin lesions (POR = 4.8, 95% CI = 2.1–10.9); and low knowledge of diphtheria (POR = 2.4, 95% CI = 1.2–4.7). Contact with a case of diphtheria; sharing a bed or bedroom; sharing utensils, cups, and glasses; infrequent bathing; and low parental education were associated with diphtheria in multiple studies. Evidence for other factors was inconclusive. The quality of evidence was low or very low for all the risk factors.

**Conclusions:**

Findings from the review suggest that countries seeking to control diphtheria need to strengthen surveillance, improve vaccination coverage, and increase people's knowledge of the disease. Future research should focus on understudied or inconclusive risk factors.

## Introduction

Diphtheria is a vaccine-preventable disease primarily caused by *Corynebacterium diphtheriae* [[1], [2], [3]]. Transmission of *C. diphtheriae* generally occurs via contact with respiratory droplets or skin secretions of an infected individual. An infected person generates an estimated 2–4 cases [4]. Diphtheria can cause complications, including respiratory insufficiency, myocarditis, and neuritis [[4], [5], [6], [7]]. The case fatality rate is 5–10% [[5], [6], [7]], but can reach 29% among untreated, unvaccinated patients [4].

Historically, diphtheria was a major cause of mortality, especially among children. In the 1970s, before the widespread use of vaccines, one million diphtheria cases (including 50,000–60,000 deaths) were estimated to occur annually in low- and middle-income countries (LMICs) [8]. Following the launch in 1974 by the World Health Organization (WHO) of the Expanded Programme on Immunization, diphtheria incidence decreased by over 90% between 1980 and 2000 [[8], [9], [10]]. After a period of relative stability, there was a recrudescence of the disease, with 22,625 cases reported globally in 2019 – the highest number of cases since 1996, when 28,624 cases were notified [9]. The resurgence of diphtheria has been fueled by large outbreaks in Bangladesh, Haiti, Venezuela, and Yemen [4,9,11]. These outbreaks are a reminder of the continued threat posed by the disease to communities worldwide.

The characteristics of diphtheria have been described thoroughly, including its prevalence, routes of transmission, and incubation period [4,8,10,12]. However, to date, there has not been a comprehensive systematic review of its risk factors or meta-analysis of the strength of association between these factors and diphtheria. Increased knowledge on this subject would allow a more accurate detection of at-risk populations. It would also help develop more effective interventions, ultimately reducing the burden of disease. Therefore, we aimed to conduct a systematic review and meta-analysis that addresses the following two questions: what are the modifiable risk factors for diphtheria? What is the strength of association between these factors and the disease?

## Methods

This systematic review and meta-analysis was conducted in accordance with the Preferred Reporting Items for Systematic Reviews and Meta-Analysis (PRISMA) statement (Appendix 1) [13]. Before starting the study, the protocol was registered with PROSPERO (CRD42019128741) [14]. The authors received no specific funding for this study.

### Search strategy

A literature search was conducted using EMBASE, MEDLINE, PubMed, and Web of Science. To identify grey literature, we consulted Gavi publications library, OpenGrey, and WHO Library Information System (WHOLIS). All databases were searched from inception until January 2023. No restrictions were applied. Native speakers translated studies in non-English languages.

The search strategy combined Medical Subject Headings (MeSH), free-text terms, and keywords related to diphtheria and risk factors. The strategy was developed for MEDLINE and adapted for EMBASE, PubMed, and Web of Science (**Appendix 2**). Only the term “diphtheria” was used for other databases.

### Eligibility criteria

To be included, studies had to meet two criteria: report on diphtheria cases, as described by the authors – including individuals with a laboratory or clinical diagnosis of diphtheria [15]; and, present estimates of association for at least one potential risk factor or sufficient data to calculate these. In this study, a risk factor was defined as a modifiable variable associated with an increased likelihood of diphtheria. Eligible measures of association were odds ratios (ORs), risk ratios (RRs), alongside regression and correlation coefficients.

Studies that focused on non-modifiable risk factors for the disease (e.g., age, sex, and ethnicity) or on risk factors for diphtheria severity or mortality were deemed to be outside the scope of the review. Single case reports, qualitative studies, commentaries, editorials, economic analyses, letters, literature reviews, and animal studies were also excluded.

### Study selection

A single reviewer (JI) exported all studies to EndNote X9 (Clarivate Analytics, Philadelphia, USA). The study selection then followed three stages. Firstly, one reviewer (JI) screened the titles of all papers to eliminate those that were clearly irrelevant to the review. Secondly, two reviewers (JI and BA) independently screened the abstracts of retained papers, excluding those that did not meet the eligibility criteria. Thirdly, both reviewers independently examined the full text of each study deemed potentially relevant based on the abstracts. References of included studies were screened to identify other relevant publications. Disagreements were resolved by discussion between the two reviewers and, when needed, referred to a third person (SL).

### Data extraction

Two reviewers (JI and BA) independently extracted data from included studies using a standardized Excel spreadsheet (Microsoft, Redmond, United States). For each study, the following variables were recorded: last name of the first author, year of publication, country where the study was conducted, study design, number of cases, number of controls, definition of cases, definition of controls, age range of the sample, diagnostic method of diphtheria infection, considered confounders, data collection technique, and effect estimates. If studies did not report effect estimates, available data in those papers were used to calculate crude ORs from two-by-two tables. Where multiple estimates were provided, those adjusted for the most confounders were selected. The fully adjusted estimates were the most homogenous in terms of included confounders. This choice was made to reduce the impact of confounding as a source of heterogeneity. A sensitivity analysis was conducted to evaluate the impact of adjustment for confounding on the identified risk factor with the most evidence (i.e., incomplete vaccination). A sensitivity analysis was also performed to assess the impact of excluding the one preprint article on the pooled estimate for another risk factor (i.e., contact with a diphtheria case).

### Quality assessment

Two reviewers (JI and WE) independently evaluated the methodological quality of case-control, cohort, and cross-sectional studies using the Newcastle-Ottawa Scale (NOS) [16,17]. The evaluation was based on three main domains: selection of study groups, comparability between cases and controls, and ascertainment of the outcome of interest. For case-control and cohort studies, eight items were assigned one or two points, for a maximum score of nine. For cross-sectional studies, seven items received one or two points, for a maximum score of 10. Each score denoted a different level of quality: low (0–3), moderate (4–7), and high (≥8).

The quality of evidence for each risk factor across the studies was assessed using the Grading of Recommendations Assessment, Development, and Evaluation (GRADE) criteria [18,19]. The quality was rated very low, low, moderate, or high. In line with the GRADE approach, evidence from observational studies was initially rated by default as low quality due to residual confounding. Evidence could then be downgraded based on the consideration of five domains: risk of bias (as indicated by the NOS score), imprecision (if the confidence intervals were wide or the total population size was <400 – a threshold “rule-of-thumb” value), inconsistency (if there was variability in the direction or magnitude of effect across studies), indirectness (if there were factors relating to the study population that could limit generalizability), and publication bias (if there was a high probability of unreported studies). Criteria for upgrading evidence included a dose–response relationship or large effect size (OR ≥ 2 or OR ≤ 0.5).

For NOS and GRADE assessments, discrepancies between the two reviewers were resolved by consensus and, if needed, by adjudication of a third reviewer (SL).

### Data analysis

Included studies were summarized in a qualitative synthesis. As diphtheria is a relatively rare event, ORs and RRs were considered equivalent [20,21]. A meta-analysis was conducted if two or more studies captured the same risk factor but from different samples. The random effects model was chosen for the analysis due to variations in study methods and populations [22]. Original data from the studies were combined to calculate pooled odds ratios (PORs) with 95% confidence intervals (CIs) using the Mantel-Haenszel method [23]. If studies only presented adjusted data, PORs were estimated using the generic inverse weighted method [24].

Forest plots were generated to display individual and global estimates. Heterogeneity among studies was assessed using the *I*^*2*^ statistic [25]. Values of 25%, 50%, and 75% revealed low, medium, and high heterogeneity, respectively. All analyses were performed using Review Manager 5.3 (Cochrane Collaboration, Copenhagen, Denmark).

## Results

**Fig. 1:**
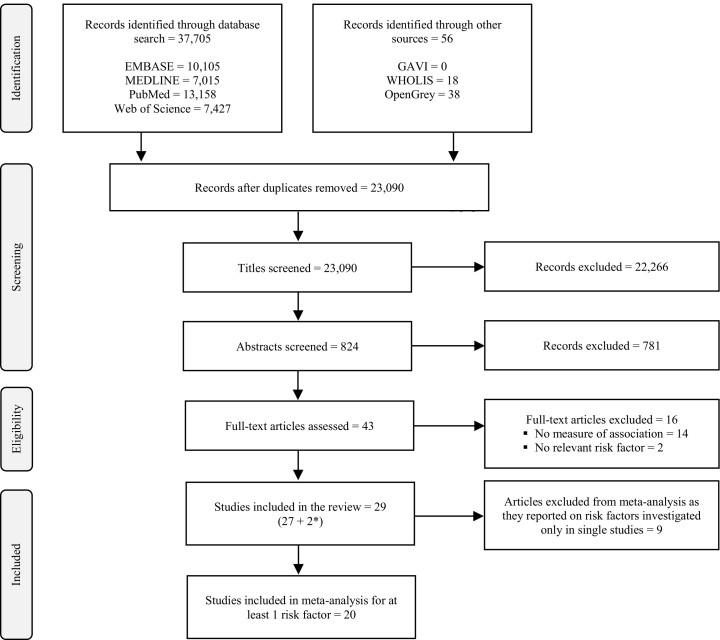
Search and study selection process. *Additional papers included following hand searching of reference lists.

After the initial search, 43 publications were selected for full-text review (Fig. 1). Fourteen were excluded as they did not provide measures of association and two because the investigated exposures were irrelevant. In addition to the remaining 27 publications, two other references were identified by hand searching. Twenty-nine papers were included in the review and 20 in the meta-analysis. Nine papers were excluded from the meta-analysis as they reported on risk factors that had not been investigated in other studies; therefore, their results could not be pooled with data from other publications.

### Study characteristics

Table 1 describes the characteristics of the 29 included studies. Overall, there were 14 case-control studies, eight cross-sectional studies, six ecological studies, and one cohort study. All non-ecological studies were of moderate to high quality (**Appendix 3**). The studies covered a period from 1907 to 2021, with most of the studies (55%) focusing on outbreaks that occurred prior to the year 2000. Only one study reported on an outbreak with cases identified both before and after 2000. The studies were conducted in five WHO regions: Europe (nine), Americas (eight), South-East Asia (six), Eastern Mediterranean (three), and Western Pacific (three). No study was done in Africa.

**Table 1:** Characteristics of included studies (*n* = 29).

Reference	WHO region	Country	Study period	Cases (n)	Controls (n)	Age (years)	Method of diagnosis	Statistical analysis	Considered confounders	NOS score
Case-control studies										
Allam 2016 [26]	SEAR	India	2013	63	63	10–45	Clinical	Multivariate logistic regression	Age, day of healthcare visit, location	7
Bisgard 2000 [27]	EUR	Russia	1991–1992	217	2168	0–14	Clinical or laboratory	2 × 2 cross-tabulation	Age, location	8
Bitragunta 2008 [28]	SEAR	India	2003–2006	123	123	0–10	Laboratory	Conditional logistic regression	Age, location	7
Brennan 2000 [29]	EUR	Russia	1995–1996	39	117	40–49	Laboratory	Conditional logistic regression	Age, location	9
Chen 2018 [30]	EUR	Ukraine	1992	262	517	0–14	N/A	2 × 2 cross-tabulation	Age, location	7
Faria 1971 [31]	AMR	Brazil	1969	27	24	1–12	N/A	2 × 2 cross-tabulation	Age, location, sex	4
Husada 2018 [32]	SEAR	Indonesia	2011–2015	27	108	0–18	Laboratory	Multivariate logistic regression	Age, location	6
Jones 1985 [33]	EMR	Yemen	1981–1982	47	94	N/A	Clinical or laboratory	Multivariate logistic regression	Age, location, sex	6
Murakami 2010 [34]	WPR	Vietnam	2005–2006	88	352	1–32	Clinical	Multivariate logistic regression	Age, location, sex	7
Nassar 2021 [35]	EMR	Yemen	2019	76	152	N/A	Clinical or laboratory	Multivariate logistic regression	Age, location, sex	8
Quick 2000 [36]	EUR	Georgia	1995–1996	218	408	0–75	Clinical	Multivariate logistic regression	Age, location, sex	6
Ramdan 2018 [37]	SEAR	Indonesia	2017–2018	18	19	1–10	N/A	Multivariate logistic regression	Age, location, sex	7
Sein 2016 [38]	WPR	Laos	2012–2013	42	79	0–45	Clinical	Bivariate logistic regression	Age, location, sex	8
Vitek 1999 [39]	EUR	Russia	1994–1996	58	306	6–8	Laboratory	Conditional logistic regression	Age, class of attendance, location	8

Cohort study										
Chandra 1973 [40]	SEAR	India	1971	14	114	0–5	Laboratory	2 × 2 cross-tabulation	N/A	6

Cross-sectional studies										
Belsey 1969 [41]	AMR	United States	1966–1967	249	3246	N/A	Laboratory	2 × 2 cross-tabulation	N/A	4
Harnisch 1989 [42]	AMR	United States	1974–1975	7	888	N/A	Laboratory	2 × 2 cross-tabulation	N/A	8
Kalapothaki 1984 [43]	EUR	Greece	1980	28	790	6–12	Laboratory	2 × 2 cross-tabulation	N/A	7
Kitamura 2023 [44]	WPR	Vietnam	2019	27	1189	1–55	Laboratory	2 × 2 cross-tabulation	Age	9
Marcuse 1973 [45]	AMR	United States	1970	131	978	N/A	Clinical or laboratory	2 × 2 cross-tabulation	N/A	8
Miller 1972 [46]	AMR	United States	1970	104	202	N/A	Laboratory	2 × 2 cross-tabulation	N/A	8
Ohuabunwo 2005 [47]	EUR	Latvia	2005	24	281	N/A	Laboratory	Logistic regression	N/A	9
Trichopoulos 1972 [48]	EUR	Greece	1970–1971	124	83	6–13	Laboratory	2 × 2 cross-tabulation	N/A	8

Ecological studies										
Coleman 2018 [49]	AMR	United States	1907–1923	221,018	0	N/A	N/A	Linear regression	N/A	–
Dureab 2019 [50]	EMR	Yemen	2017	1294	0	N/A	Clinical	Multivariate logistic regression	N/A	–
Ikejezie 2022 [51]	AMR	Haiti	2014–2021	392	0	N/A	Clinical or laboratory	Geographically weighted regression	N/A	–
Izza 2015 [52]	SEAR	Indonesia	2010–2011	968	0	N/A	N/A	Correlation tests	N/A	–
Podavalenko 2018 [53]	EUR	Ukraine	1985–2012	21,348	0	N/A	N/A	Binary logistic regression	N/A	–
Quesada 1979 [54]	AMR	United States	1970	201	0	N/A	N/A	Stepwise logistic regression	N/A	–

N/A: Not available

Twenty-seven studies were written in English, one in Portuguese, and one in Bahasa Indonesia. Sample sizes (cases and controls combined) ranged from 37 to 221,018 (median = 364). Nine studies involved only children (age range = 0–14 years); one study only involved adults (age range = 40–49 years); six studies involved both children and adults; and thirteen studies did not indicate the age range of the sample. Where reported, the median proportion of male participants was 51% (range = 30–93%). Infected individuals were diphtheria cases (20), asymptomatic carriers (six), or a combination of the two (three). Diphtheria was ascertained by isolation of *C. diphtheriae* in culture (12), clinical examination (six), and using either one of the two methods (five); six studies did not specify the method of diagnosis.

### Potential risk factors

Ninety-five potential risk factors were abstracted from the 29 articles (Appendix 4). Altogether, 32 factors were associated with diphtheria in at least one study (Table 2; **Appendix 5**). Of these, five were related to vaccination or contact with cases, five to underlying conditions, 10 to knowledge and behaviour, two to socioeconomic status, and 10 were population-level factors.

**Table 2:** Factors investigated for which at least one study found an association with diphtheria.

Theme and risk factor	No.	Meta-analysis	Risk estimate (95% CI or *p* value), *I*^*2* Ref.^	GRADE score
Vaccination or contact with cases				
Incomplete vaccination (<3 doses)	18	Yes	POR = 2.2 (1.4–3.4), *I*^*2*^ = 77% [27,28,30,[32], [33], [34], [35], [36], [37], [38],^41^,[43], [44], [45], [46],^48^,^55^] N/A [47]	Moderate
No booster vaccination in last five years	4	Yes	POR = 3.6 (0.5–24.0), *I*^*2*^ = 67% [29,39] N/A [34,47]	Very low
Partially vaccinated sibling	1	No	OR = 4.1 (1.8–9.0) [31]	Very low
Contact with a diphtheria case	5	Yes	POR = 5.0 (0.8–31.8), *I*^*2*^ = 75% [33,[35], [36], [37]] N/A [34]	Low
Contact with a person with skin lesions	3	Yes	POR = 4.8 (2.1–10.9), *I*^*2*^ = 0% [33,36] N/A [34]	Low

Underlying conditions				
Having tonsils	2	Yes	POR = 2.0 (0.4–10.0), *I*^*2*^ = 83% [36,48]	Very low
Recent sore throat	2	Yes	POR = 1.8 (0.8–4.0), *I*^*2*^ = 71% [36,48]	Very low
History of chronic illness	2	No	OR = 2.1 (1.2–3.8) [36] N/A [34]	Very low
History of eczema	1	No	OR = 3.4 (1.2–9.9) [36]	Very low
Recent fever with myalgia	1	No	OR = 2.7 (1.3–5.5) [36]	Very low

Knowledge and behaviour				
Sharing utensils, cups, glasses	4	Yes	POR = 1.7 (1.0–2.9), *I*^*2*^ = 62% [35,36,47] N/A [34]	Very low
Sharing a bed or bedroom	4	No	POR = 1.3 (0.6–3.0), *I*^*2*^ = 76% [35,36,38,44]	Very low
Low diphtheria knowledge	3	Yes	POR = 2.4 (1.2–4.7), *I*^*2*^ = 20% [26,32,37]	Low
Bathing once a day or less	2	No	OR = 1.7 (1.04–2.9) [34] *p* = 0.40 ^44^	Very low
Bathing once a week or less	1	No	OR = 2.6 (1.3–5.2) [36]	Very low
Alcohol abuse	2	No	OR = 48.8 (27.2–87.6) [42] OR = 0.7 (0.3–1.7) [36]	Very low
Travel history to area with diphtheria	2	Yes	POR = 2.2 (0.1–34.1), *I*^*2*^ = 84% [35,37]	Very low
Belief that vaccines are ineffective	1	No	OR = 4.0 (1.2–13.5) [26]	Very low
Consumption of factory-made yoghurt	1	No	OR = 14.9 (*p* = 0.003) [33]	Very low
Obtaining water from a wheeled carrier	1	No	OR = 28.4 (*p* = 0.008) [33]	Very low

Socioeconomic status				
Low maternal education	3	Yes	POR = 1.5 (0.6–3.8), *I*^*2*^ = 53% [32,35,36]	Low
Low paternal education	2	No	POR = 1.7 (0.2–14.9), *I*^*2*^ *=* *88%* [32,35]	Low

Population-level factors				
Population density	4	No	ß = 0.04 (*p* < 0.001) [53]β = 0.004 (*p* > 0.05) [54] *r* = 0.002–0.07 (*p* > 0.05) [52]β = −0.001 (p > 0.05) [51]	Very low
Vaccination coverage	4	No	ß = −0.04 (*p* = 0.001) [53] *r* = 0.42 (*p* = 0.008); *r* = 0.22 (*p* = 0.183) [52] OR = 1.04 (1.01–1.06) [50]β = 0.177 (*p* > 0.05) [51]	Very low
Degree of urbanization	2	No	ß = 0.006 (*p* < 0.01) [51] N/A [53]	Very low
Female literacy	1	No	ß = −0.024 (*p* < 0.001) [51]	Very low
Morbidity rate in the urban population	1	No	ß = 0.15 (p = 0.001) [53]	Very low
Ongoing conflict	1	No	OR = 11.2 (1.3–97.7) [50]	Very low
Percentage of people below poverty line	1	No	ß = 0.02 (*p* < 0.05) [54]	Very low
Population growth rate	1	No	ß = −0.23 (*p* < 0.001) [53]	Very low
Sulfur dioxide air levels	1	No	ß = 0.23 (*p* < 0.001) [53]	Very low
Tuberculosis cases	1	No	ß = 0.03–0.14 (*p* < 0.05); β = 0.03 (p = 0.41) [49]	Very low


ß, beta coefficient; OR, odds ratio; POR, pooled odds ratio; r, correlation coefficient.

N/A, effect estimate not available.

#### Vaccination or contact with cases

**Fig. 2:**
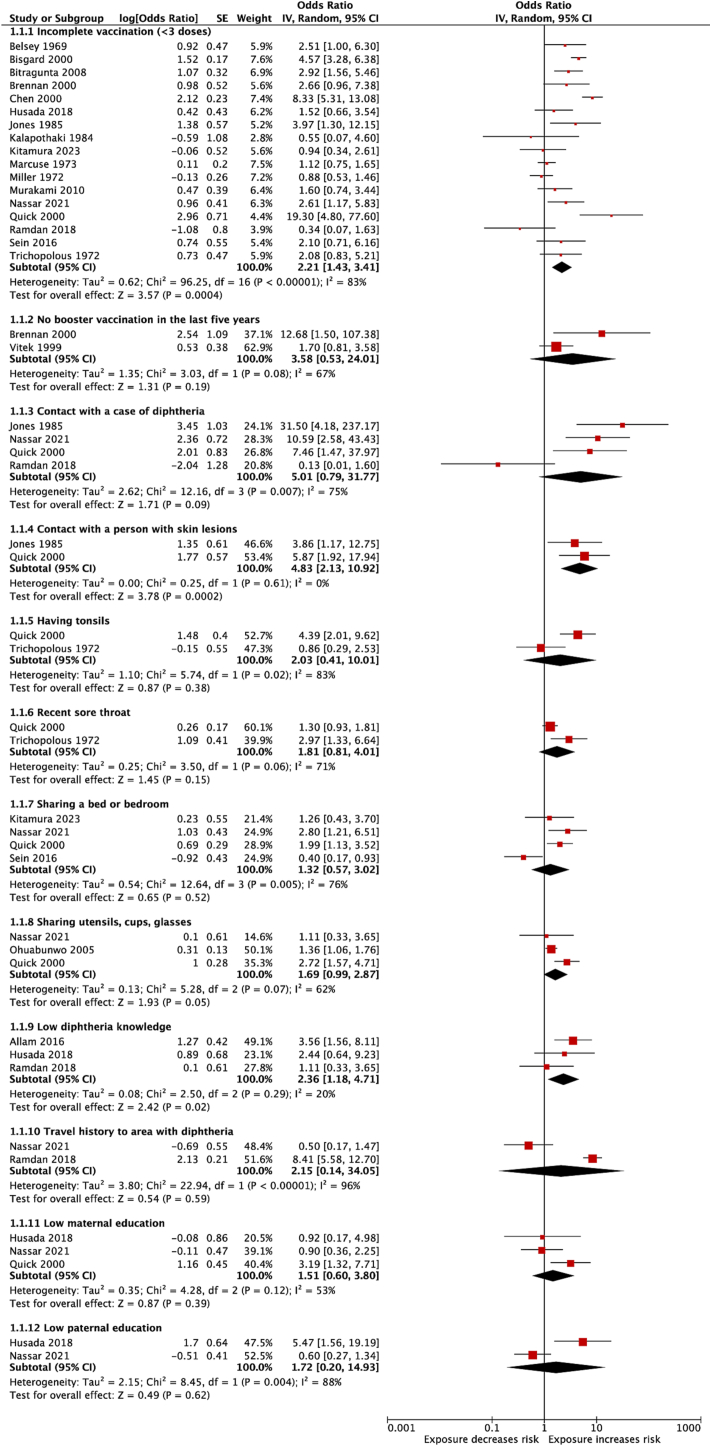
Risk of diphtheria associated with specific risk factors.

Meta-analysis of 17 studies [27,28,30,[32], [33], [34], [35], [36], [37], [38],^41^,[43], [44], [45], [46],^48^,^55^] showed that incomplete vaccination (i.e., having received less than three primary doses of the diphtheria vaccine) doubled the odds of diphtheria (POR = 2.2, 95% CI = 1.4–3.4) (Fig. 2). From the sensitivity analysis, after excluding studies that did not adjust for potential confounders, the conclusion remained the same – with the direction of effect concordant with the main meta-analytic results (**Appendix 6**).

Meta-analysis of two studies conducted in Russia in the mid-1990s [29,39] found no increased risk of diphtheria associated with not having received a booster vaccination in the preceding five years (POR = 3.6, 95% CI = 0.5–24.0); nevertheless, the point estimate was relatively high and the studies had limited power. Two other studies [34,47] also reported no increased risk of diphtheria associated with time since last booster vaccination; however, these studies did not present effect estimates, nor did they specify the intervals used for the estimations [34,47]. Finally, data from a study conducted in Brazil in 1969 pointed towards an increased risk of diphtheria associated with having a partially vaccinated sibling (OR = 4.1, 95% CI = 1.8–9.0) [31].

Meta-analysis of four studies (two from Yemen [33,35], one from Georgia [36], one from Indonesia [37]) revealed no increased risk of acquiring diphtheria following contact with a diphtheria case (POR = 5.0, 95% CI = 0.8–31.8). However, the pooled estimate appeared to be skewed by one study – a preprint article, which also reported outlier values for other risk factors (e.g., incomplete vaccination, low diphtheria knowledge). After removing this study from the meta-analysis, contact with a diphtheria case was associated with an increased risk of diphtheria (**Appendix 6**). Meta-analysis of two [33,36] of the four studies suggest that contact with a person with skin lesions (POR = 4.8, 95% CI = 2.1–10.9) does increase the risk of diphtheria. A fifth study from Vietnam [34] found no increased risk of infection related to the two exposures; nevertheless, the study was excluded from the meta-analysis as no effect estimates or data to calculate these were provided.

#### Underlying conditions

Meta-analysis of two studies from Georgia [36] and Greece (which did not adjust for potential confounders) [48] found that neither having tonsils (POR = 2.0, 95% CI = 0.4–10.0) or having a recent sore throat (POR = 1.8, 95% CI = 0.8–4.0) increased the risk of diphtheria. The study from Georgia also reported a doubling of the odds of infection related to having a chronic illness (OR = 2.1, 95% CI = 1.3–5.5) [36], while a study from Vietnam found no association with the exposure [34]; nonetheless, the latter study did not provide effect estimates or data to calculate these. Additionally, in the study from Georgia [36], both history of eczema (OR = 3.4, 95% CI = 1.2–9.9) and recent fever with myalgia (OR = 2.7, 95% CI = 1.3–5.5) were associated with diphtheria.

#### Knowledge and behaviour

Meta-analysis of three recent studies from India [26] and Indonesia [32,37] showed that the odds of disease more than doubled for people with low knowledge of diphtheria (as assessed by a questionnaire) (POR = 2.4, 95% CI = 1.2–4.7). The study from India also reported an increased risk of diphtheria associated with believing that vaccines do not prevent diseases (OR = 4.0, 95% CI = 1.2–13.5) [26].

Meta-analysis of three studies from Georgia [36], Latvia (which did not adjust for potential confounders) [47], and Yemen [35] did not identify an increased risk of diphtheria associated with sharing utensils, cups, or glasses (POR = 1.7, 95% CI = 1.0–2.9). A fourth study from Vietnam [34] found no increased risk of infection related to this exposure; nevertheless, the study was excluded from the meta-analysis as it did not provide effect estimates or data to calculate these. Meta-analysis of four studies from Georgia [36], Laos [38], and Yemen [35] also found that sharing a bed or bedroom with other people did not increase the risk of diphtheria (POR = 1.3, 95% CI = 0.5–3.9).

Results relating to infrequent bathing were discordant. Two studies from Vietnam examined the association between diphtheria and bathing once a day or less (as opposed to twice a day or more). While one study found no association between the two variables (*p* = 0.40) [44], the other found that bathing once a day or less increased the odds of infection (OR = 1.7, 95% CI = 1.04–2.9) [34]. Furthermore, a study from Georgia found that bathing less than once a week also increased the risk of diphtheria (OR = 2.6, 95% CI = 1.3–5.2) [36].

Meta-analysis of two studies from Indonesia [37] and Yemen [35] found no increased risk of infection associated with travel to an area with diphtheria cases (POR = 1.3, 95% CI = 0.5–3.9). A study conducted in Yemen in the 1980s reported an increase in the odds of diphtheria associated with obtaining water from a wheeled carrier (i.e., a person who carries water obtained from wells to people's houses) (OR = 28.4, *p* = 0.008) and consumption of factory-made yoghurt (OR = 14.9, *p* = 0.003) [33].

The study from Georgia also found that having ≥14 alcoholic drinks per week did not increase the risk of diphtheria (OR = 0.7, 95% CI = 0.3–1.7) [36]; by contrast, data from a study done in the 1970s in the United States (which did not adjust for potential confounders) indicated a higher risk of disease for “heavy consumers of alcoholic beverages” (OR = 48.8, 95% CI = 27.2–87.6) [42].

#### Socioeconomic status

The only socioeconomic indicators that showed a potential association with diphtheria were related to the education level of parents. Meta-analysis of three studies from Indonesia [32], Georgia [36], and Yemen [35] suggested no increased odds of diphtheria associated with having a mother with primary education or less (POR = 1.5, 95% CI = 0.6–3.8). Similarly, meta-analysis of two studies from Indonesia [32] and Yemen [35] found no increased risk of diphtheria for children whose fathers had primary education or less (POR = 1.7, 95% CI = 0.2–14.9).

#### Population-level factors

Above-mentioned risk factors were assessed at the individual level. Instead, the following factors were examined at the population level in ecological studies comparing diphtheria incidence or case counts across different areas of the same country.

Four studies from Haiti [51], Indonesia [52], Ukraine [53], and Yemen [50] examined the association between diphtheria and vaccination coverage. The study from Indonesia identified a positive correlation between vaccination level and case count during the first year of a diphtheria outbreak (*r* = 0.42, *p* = 0.008); however, the following year, the two variables were no longer correlated (*r* = 0.22, *p* = 0.183). The study from Ukraine found a negative association between vaccination coverage and diphtheria incidence in six oblasts (ß = -0.04, *p* = 0.001). This result indicated that a unit increase in vaccination coverage was associated with a 4% average decrease in incidence rates, all else being equal. The study from Yemen showed that vaccination coverage affected the odds of a diphtheria outbreak (OR = 1.04; 95% CI =1.01–1.06, *p* = 0.002). In contrast with other articles, the study from Haiti did not find an association between diphtheria incidence and vaccination coverage (ß = 0.177; *p* > 0.05). The authors of the study explained that weaknesses in the country's immunization information systems and inaccuracies in the vaccination coverage estimates may have contributed to the observed lack of association between the two variables.

The association between diphtheria and population density was examined by four studies from Haiti [51], Indonesia [52], Ukraine [53], and the United States [54]. Only the study from Ukraine found a positive association between diphtheria incidence and population density (ß = 0.04, *p* < 0.001) [53], whereas the other studies reported no association between the two variables.

A study that analyzed historical records from the early 20th century in the United States found a positive association between diphtheria case counts and reports of tuberculosis in four of five examined cities (β = 0.06–0.14, *p* < 0.05); no association was observed in the other city (β = 0.03, *p* = 0.41) [49].

Seven population-level factors were associated with diphtheria in single studies from Haiti [51], Ukraine [53], the United States [54], and Yemen [50]: female literacy (β = −0.02, *p* < 0.001) [51], morbidity rate in the urban population (β = 0.15, *p* = 0.001) [53], population growth rate (β = −0.23, p < 0.001) [53], presence of an ongoing conflict (OR = 11.2, 95% CI = 1.3–97.7) [50], sulfur dioxide air levels (β = 0.23, p < 0.001) [53], and percentage of people below the poverty line (ß = 0.02, *p* < 0.05) [54].

#### Quality of evidence

The quality of evidence for the 32 identified risk factors varied from moderate to very low. Evidence for all risk factors was initially rated of low quality due to the observational nature of the included studies. Only the evidence for incomplete vaccination was upgraded from low to moderate given the strength of association with diphtheria observed in the meta-analysis and sensitivity analysis. Evidence for the other risk factors was often downgraded from low to very low due to concerns related to imprecision as most of the estimates came from a few studies, which in several instances had small sample sizes. Evidence for numerous risk factors was also downgraded due to inconsistencies in the direction of the effect across studies. The risk of methodological bias was considered serious only for population-level factors, whose evidence came from ecological studies. No risk of publication bias was detected for the 32 risk factors. Appendix 4 illustrates the full assessment of the quality of evidence.

## Discussion

This systematic review revealed several factors associated with an increased risk of diphtheria: incomplete vaccination, contact with a person with skin lesions, and low knowledge of diphtheria. Other factors showed potential associations with the disease in multiple studies, including no booster vaccination in the last five years; contact with a case of diphtheria; sharing a bed or bedroom; sharing utensils, cups, and glasses; and infrequent bathing. The quality of evidence for the identified risk factors was rated as low or very low – except for incomplete vaccination, whose evidence was judged of moderate quality. Such a finding is not uncommon. Many systematic reviews following Cochrane or WHO guidelines are based on observational evidence rated as low quality [56,57]; however, these studies are very important in public-health decision-making where randomized controlled trials are not available or ethical.

While GRADE offers a viable approach for evaluating the quality of evidence, it may not be completely suitable for assessing the quality of evidence relating to nonmodifiable risk factors, such as those identified in this systematic review. This is because evidence for this type of risk factor generally comes from observational studies, whose evidence is always initially rated as low-quality using GRADE – irrespective of the study design or the methodological quality of the studies. When the entirety of the evidence comes from observational studies, it may be advisable to not consider this as a criterion for quality assessment.

In the sensitivity analysis, incomplete vaccination remained associated with an increased risk of diphtheria after removing studies that did not adjust for potential confounders. This finding was consistent with studies of other vaccine-preventable diseases [[58], [59], [60], [61]], which showed a higher risk of infection for partially vaccinated individuals. This finding was also in line with results of another review [62], which reported that two primary vaccine doses produced lower protective levels of diphtheria antibodies compared with three doses. The importance of vaccines was further corroborated by other studies included in the present review [53,63], which pointed towards an association between diphtheria and vaccination coverage.

In the meta-analysis, the POR for not having received a booster vaccination in the last five years suggested a potential increased risk of diphtheria. The result might have been inconclusive due to low power and heterogeneity of the study populations. Five years might have also been insufficient to identify time since the last booster as a risk factor, especially in children. Among adults, for which there were fewer studies, this may be a strong risk factor, as suggested by one of the included studies [29]. Previous studies [[64], [65], [66], [67]] found that immunity to diphtheria decreases as age increases. Further research could provide valuable evidence to inform vaccination guidelines for adults, which currently vary extensively worldwide [68,69].

Exposure to an infected person is an established risk factor for various vaccine-preventable diseases [58,60,[70], [71], [72], [73]]. Hence, we anticipated to find that individuals who had contact with a diphtheria case or a person with skin lesions (a potential proxy for cutaneous diphtheria) had a higher risk of infection compared to those who did not. These results support current surveillance guidelines that recommend the prompt identification, monitoring, and implementation of preventive measures for close contacts of diphtheria cases [3,5,6].

The association between diphtheria and low knowledge of the disease was unsurprising. The increased risk of diphtheria associated with believing that vaccines do not prevent diseases, as reported by one of the included studies, was also expected. Past studies have shown that knowledge of vaccine-preventable diseases and attitudes towards vaccination influence vaccine uptake and adoption of other protective measures [[74], [75], [76]]. It is unclear whether antivaccination attitudes have been increasing in countries that are reporting diphtheria cases. Nonetheless, the recent rise of the internet and social media has certainly created unprecedented opportunities for antivaccination messages and false health information to spread virally, potentially shaping people's knowledge, attitudes, and actions [77,78].

The increased risk of diphtheria associated with sharing utensils, cups, and glasses; bathing infrequently; and obtaining water from a wheeled carrier (a possible indicator of a lack of access to clean water and sanitation) suggested that poor hygiene may play a role in contracting the disease. While the exact mechanism through which such practices heighten the likelihood of disease is not fully understood, it is conceivable that they might increase susceptibility to *C. diphtheriae* colonization [36]. Our results support those of previous systematic reviews, which concluded that adherence to good hygiene practices lowers the risk of respiratory infections [79,80].

Incomplete vaccination has often been associated with low parental education [81,82]. This review also revealed studies indicating a potential relation between education and diphtheria, with children whose parents had low educational levels appearing to be at an increased risk of infection. The pathway behind this association is unclear. Less educated parents may have lower literacy skills that makes them less receptive to health information [[83], [84], [85]]. Less educated parents may also have lower communication abilities, which decrease their capacity to navigate the healthcare system to have their children vaccinated [83,84,86]. Finally, less educated parents may simply live in poorer areas where the risk of acquiring diphtheria is higher due to reduced access to healthcare, lower vaccination coverage, and worse sanitary conditions [83,87,88]. This last hypothesis seems to be supported by the link identified in one of the studies included in this review between the share of people living below the poverty line and diphtheria incidence.

## Implications

Given the strength of association between diphtheria and incomplete vaccination alongside the consistency of our findings with the existing body of knowledge on vaccine-preventable diseases, additional observational studies on this risk factor would have limited value – except for better characterizing its association with diphtheria. Further research on this subject should, therefore, focus on other factors presenting a possible link with diphtheria and on factors overlooked by previous studies. Overlooked factors include those related to healthcare (e.g., availability of health workers, proximity to health centres, possession of health insurance), which have shown associations with vaccination status [82,89,90]. Finally, the risk factors presented in this paper could be used to adjust for confounding in future studies that investigate potential risk factors for diphtheria.

By identifying the main modifiable diphtheria risk factors, this review provided a basis for detecting those most vulnerable to the disease and an agenda for public health action. The observed high risk of infection following exposure to a person with skin lesions, alongside evidence in some of the included studies of an increased risk of infection after contact with a diphtheria case, underscored the importance of early case finding and contact tracing. These activities entail the existence of adequate means for laboratory testing. Recent studies highlighted major gaps in diphtheria diagnostics in Europe and in the Western Pacific, including a lack of capacity for molecular typing, insufficient testing equipment, and inadequate training of laboratory personnel [91,92]. These challenges are likely present in other parts of the world. Tackling them is crucial for controlling diphtheria.

The overwhelming evidence of a link between diphtheria and incomplete vaccination reinforced the need for countries to achieve timely vaccination with a complete primary series followed by booster doses, as recommended by the WHO [8]. In the 1990s, countries of the former Soviet Union succeeded in controlling widespread diphtheria epidemics by vaccinating >140 million adults and adolescents alongside millions of children [93]. Since such mass interventions might be difficult for some of the countries currently affected by diphtheria, other solutions should be explored. The recent experience of many LMICs (some of which are reporting high diphtheria rates; e.g., Haiti and India) with delivering the human papillomavirus vaccine in schools showed that large numbers of children can be reached through school-based vaccination [94,95].

Efforts should be made to increase people's knowledge of diphtheria and improve understanding of how to prevent the disease. As healthcare professionals are generally considered the most trusted source of health information [[96], [97], [98], [99]], they should be encouraged to inform patients about diphtheria and the importance of personal hygiene and vaccines. Furthermore, activities should be directed towards implementing effective social mobilization strategies. Polio eradication initiatives in Afghanistan, India, Nigeria, and Pakistan have demonstrated that community health workers can help raise public awareness about vaccination and support surveillance activities [[100], [101], [102], [103]].

In this paper, we identified a lack of studies from Africa, despite some countries in this region having reported high rates of diphtheria in recent years. This might reflect the fact that diphtheria is a relatively rare disease that mainly affects LMICs, where research capacity is sometimes limited [104,105]. Furthermore, previous studies have shown that the fees that journals charge to publish articles open access can also pose a barrier for researchers and research institutions in LMICs [106,107]. The dearth of studies on diphtheria might also be partly due to disparities in research priorities, with higher attention given to diseases perceived to have greater public health impact, such as HIV/AIDS, malaria, and tuberculosis. Governments and donor agencies should allocate adequate funding for the implementation of research and dissemination of findings on diphtheria to strengthen the evidence base and inform disease control efforts in countries affected by the disease. Such research is needed more than ever given the impact of the COVID-19 pandemic, which has caused the largest global disruption to routine immunization services in recent history, leaving millions of children unvaccinated or under-vaccinated against diphtheria – including in countries that had previously controlled the disease [108,109].

## Limitations and strengths

Identified risk factors emerged from a limited number of single studies, resulting in evidence of mostly low quality. The small sample sizes of many of the included studies also meant that studies were not adequately powered to reach definitive conclusions. All studies were observational; as such, they could not demonstrate causality. Certain studies did not report effect estimates for some of the investigated risk factors or data to calculate these; therefore, it was not always possible to conduct a meta-analysis. Due to the paucity of studies, we could not stratify the analysis by potential confounders (e.g., age, socioeconomic status). Included studies often varied substantially in methodology. For instance, only a minority of studies (46%) consistently used laboratory tests to diagnose diphtheria. Thus, other studies may have included individuals who did not contract the infection, possibly leading to an underestimation of the risk estimates. Differences in the methods used for the ascertainment of the exposures (e.g., surveys vs. review of medical records) may have also affected the estimates. Moreover, half of the studies did not adjust for confounders, which might have contributed to the observed inconsistencies across studies on the same risk factors. This methodological heterogeneity may partly explain the high *I*^*2*^ for some of the pooled estimates.

The high *I*^*2*^ might also be partly explained by the inclusion of studies from various contexts. The use of an extensive search strategy with clearly defined search terms and no date, geographical, or language restrictions allowed the identification and inclusion of studies from several countries spanning almost all continents, encompassing different time periods and stages of diphtheria epidemics, and covering thousands of individuals across different populations. Such diversity provided more validity to our results and enhanced the generalizability of findings. The employed search strategy helped to minimize reporting bias in our systematic review, although publication bias may still exist in the literature. Furthermore, as with every systematic review, there is a possibility that not all relevant studies were included. All included non-ecological studies were of moderate-to-high quality. The assessment of study characteristics was conducted meticulously to ensure that only papers with similar exposure definitions would be combined in a meta-analysis. As such, this review did not only provide a comprehensive synthesis of evidence on risk factors for diphtheria, but also offered summary estimates of the association between these exposures and the disease. These findings are critical since evidence-based policy and practice rely on objective measures of risk, which until now were fragmented and incomplete for diphtheria.

## Conclusions

This study identified several risk factors for diphtheria. We found moderate to low quality evidence that incomplete vaccination, contact with a person with skin lesions, and low knowledge of diphtheria increased the likelihood of disease. Contact with a case of diphtheria; sharing a bed or bedroom; sharing utensils, cups, and glasses; infrequent bathing; and low parental education also appeared to be associated with diphtheria in multiple studies. Future research should focus on risk factors that have previously been neglected or for which evidence is inconclusive.

Most of the factors identified in this review are difficult to modify. Achieving substantial reductions in the incidence of diphtheria will, therefore, require sustained efforts by countries to strengthen their laboratory capacity, improve vaccination coverage, and increase people's knowledge of the disease and prevention methods with support from health workers. While these interventions have been advocated in the past, the current resurgence of diphtheria makes their implementation as critical as ever.

## Contributions

Juniorcaius Ikejezie conceptualized and designed the study, screened articles, collected data, performed the quality assessment, carried out the statistical analysis, and wrote and revised the manuscript. Busola Adebusoye screened articles and collected data. Winifred Ekezie performed the quality assessment. Revati Phalkey, Tessa Langley, and Sarah Lewis conceptualized and designed the study, provided content expertise and methodological guidance, and reviewed the manuscript. All authors approved the final manuscript as submitted.

## Funding

This research did not receive any specific grant from funding agencies in the public, commercial, or not-for-profit sectors.

## Declaration of Competing Interest

The authors declare that they have not received any specific funding for this work, and they have no conflicts of interest related to the research presented in this article

## Figures and Tables

**Table:** 

Section / Topic	Item No.	Checklist item	Reported on page No.
**Title**			
Title	1	Identify the report as a systematic review, meta-analysis, or both	1
**Abstract**			
Structured summary	2	Provide a structured summary including, as applicable, background, objectives, data sources, study eligibility criteria, participants, interventions, study appraisal and synthesis methods, results, limitations, conclusions and implications of key findings, systematic review registration number	1
**Introduction**			
Rationale	3	Describe the rationale for the review in the context of what is already known	1-2
Objectives	4	Provide an explicit statement of questions being addressed with reference to participants, interventions, comparisons, outcomes, and study design (PICOS)	2
**Methods**			
Protocol and registration	5	Indicate if a review protocol exists, if and where it can be accessed (such as web address), and, if available, provide registration information including registration number	2
Eligibility criteria	6	Specify study characteristics (such as PICOS, length of follow-up) and report characteristics (such as years considered, language, publication status) used as criteria for eligibility, giving rationale	2
Information sources	7	Describe all information sources (such as databases with dates of coverage, contact with study authors to identify additional studies) in the search and date last searched	2
Search	8	Present full electronic search strategy for at least one database, including any limits used, such that it could be repeated	10-11
Study selection	9	State the process for selecting studies (that is, screening, eligibility, included in systematic review, and, if applicable, included in the meta-analysis)	2
Data collection process	10	Describe method of data extraction from reports (such as piloted forms, independently, in duplicate) and any processes for obtaining and confirming data from investigators	2
Data items	11	List and define all variables for which data were sought (such as PICOS, funding sources) and any assumptions and simplifications made	2
Risk of bias in individual studies	12	Describe methods used for assessing risk of bias of individual studies (including specification of whether this was done at the study or outcome level), and how this information is to be used in any data synthesis	2
Summary measures	13	State the principal summary measures (such as risk ratio, difference in means)	2
Synthesis of results	14	Describe the methods of handling data and combining results of studies, if done, including measures of consistency (such as *I*^*2*^ statistic) for each meta-analysis	2-3
Risk of bias across studies	15	Specify any assessment of risk of bias that may affect the cumulative evidence (such as publication bias, selective reporting within studies)	2
Additional analyses	16	Describe methods of additional analyses (such as sensitivity or subgroup analyses, meta-regression), if done, indicating which were pre-specified	2
**Results**			
Study selection	17	Give numbers of studies screened, assessed for eligibility, and included in the review, with reasons for exclusions at each stage, ideally with a flow diagram	3
Study characteristics	18	For each study, present characteristics for which data were extracted (such as study size, PICOS, follow-up period) and provide the citations	3-5
Risk of bias within studies	19	Present data on risk of bias of each study and, if available, any outcome-level assessment (see item 12).	3-5, 11-12
Results of individual studies	20	For all outcomes considered (benefits or harms), present for each study (a) simple summary data for each intervention group and (b) effect estimates and confidence intervals, ideally with a forest plot	5-7
Synthesis of results	21	Present results of each meta-analysis done, including confidence intervals and measures of consistency	5-7, 24-25
Risk of bias across studies	22	Present results of any assessment of risk of bias across studies (see item 15)	5, 7-8, 12-17
Additional analysis	23	Give results of additional analyses, if done (such as sensitivity or subgroup analyses, meta-regression) (see item 16)	24-25
**Discussion**			
Summary of evidence	24	Summarise the main findings including the strength of evidence for each main outcome; consider their relevance to key groups (such as health care providers, users, and policy makers)	8-9
Limitations	25	Discuss limitations at study and outcome level (such as risk of bias), and at review level (such as incomplete retrieval of identified research, reporting bias)	9
Conclusions	26	Provide a general interpretation of the results in the context of other evidence, and implications for future research	9
**Funding**			
Funding	27	Describe sources of funding for the systematic review and other support (such as supply of data) and role of funders for the systematic review	9

**Table:** 

#	Search terms
1	exp Risk Factors/
2	exp Epidemiologic Studies/
3	exp Odds Ratio/
4	exp Multivariate Analysis/
5	exp Cross-Sectional Studies/
6	exp Case-Control Studies/
7	exp Cohort Studies/
8	exp Longitudinal Studies/
9	exp Prospective Studies/
10	exp Retrospective Studies/
11	exp Logistic Models/
12	exp Prevalence/
13	exp Incidence/
14	exp Probability/
15	epidemiolog$.ti,ab
16	ecologic$.ti,ab
17	factor$.ti,ab
18	probabilit$.ti,ab
19	frequenc$.ti,ab
20	characteristic$.ti,ab
21	criteri$.ti,ab
22	prognos$.ti,ab
23	(case-control or cohort or cross-sectional or ecologic$ or retrospective or prospective or longitudinal stud$).ti,ab.
24	risk$.ti,ab.
25	predict$.ti,ab.
26	correlat$.ti,ab.
27	etiol$.ti,ab.
28	aetiol$.ti,ab.
29	prevalence$.ti,ab.
30	incidence$.ti,ab.
31	rate$.ti,ab.
32	determinant$.ti,ab.
33	odds ratio$.ti,ab.
34	associat$.ti,ab.
35	stratif$.ti,ab
36	(ROC Curve$).ti,ab.
37	discriminat$.ti,ab
38	c statistic$.ti,ab
39	(Area under the curve$).ti,ab.
40	AUC$.ti,ab
41	outcome$.ti,ab
42	indice$.ti,ab
43	algorithm$.ti,ab
44	multivaria$.ti,ab
45	model$.ti,ab
46	or/1–45
47	diphtheria$.ti,ab.
48	exp Diphtheria/
49	47 or 48
50	46 and 49

**Table:** 

#	Search terms
1	(diphtheria OR diphtheriae) AND (epidemiolog* OR ecologic* OR factor* OR probabilit* OR frequenc* OR characteristic* OR criteri* OR prognos* case-control OR cohort* OR cross-sectional OR ecologic* OR retrospective OR prospective OR longitudinal OR risk* OR predict* OR correlat* OR etiol* OR aetiol* OR prevalence* OR incidence* OR rate* OR determinant* OR odds ratio* OR associat* OR stratif* OR ROC Curve* OR discriminat* OR c statistic* OR area under the curve* OR AUC OR outcome OR indice* OR algorithm OR multivaria* OR model*)

**Table:** 

Reference	Selection				Comparability	Exposure			Score
	Adequate case definition	Cases were representative	Selection of community controls	Controls had no history of diphtheria	Comparability of cases and controls based on design or analysis	Ascertainment of exposure using a secure record or adequately blinded interviews	Same method of ascertainment for cases and controls	Same response rate for both groups	
Allam 2016	✔	✔		✔	✔✔		✔	✔	7
Bisgard 2000	✔	✔	✔	✔	✔✔	✔		✔	8
Bitragunta 2008	✔	✔	✔		✔✔		✔	✔	7
Brennan 2000	✔	✔	✔	✔	✔✔	✔	✔	✔	9
Chen 2000		✔	✔		✔✔	✔	✔	✔	7
Faria 1971					✔✔		✔	✔	4
Husada 2018	✔	✔			✔✔		✔	✔	6
Jones 1985	✔		✔		✔✔		✔	✔	6
Murakami 2010	✔	✔	✔	✔	✔✔		✔		7
Nassar 2021	✔	✔	✔	✔	✔✔		✔	✔	8
Quick 2000	✔	✔			✔✔		✔	✔	6
Ramdan 2018		✔	✔	✔	✔✔		✔	✔	7
Sein 2016	✔	✔	✔	✔	✔✔		✔	✔	8
Vitek 1999	✔	✔	✔		✔✔	✔	✔	✔	8

**Table:** 

Reference	Selection				Comparability	Outcome			Score
	Exposed cohort was representative	Non-exposed cohort was drawn from the same community as the exposed cohort	Exposure was ascertained using secure record or structured interview	There was evidence that diphtheria was absent at the start of study	The study controlled for age, location, or other factors	Outcome was assessed through an independent blind assessment or record linkage	Follow-up was sufficiently long	Follow-up was ≥ 70%	
Chandra 1973		✔			✔✔	✔	✔	✔	6

**Table:** 

Reference	Selection				Comparability	Outcome		Score
	Sample was representative	Sample size was justified	Response rate was satisfactory	Ascertainment of exposure using a validated tool	Outcome groups were comparable based on design or analysis	Outcome was assessed through an independent blind assessment or record linkage	Statistical test was described and appropriate	
Belsey 1969				✔✔		✔✔		4
Harnisch 1989	✔	✔	✔	✔✔	✔	✔✔		8
Kalapothaki 1984	✔		✔	✔✔	✔	✔✔		7
Kitamura 2023	✔	✔	✔	✔✔	✔	✔✔	✔	9
Marcuse 1973	✔	✔	✔	✔✔	✔	✔✔		8
Miller 1972	✔	✔	✔	✔✔	✔	✔✔		8
Ohuabunwo 2005	✔	✔	✔	✔✔	✔	✔✔	✔	9
Trichopoulos 1972	✔	✔	✔	✔✔	✔	✔✔		8

**Table:** 

Theme and risk factor	No.	Study design	Risk of bias	Imprecision	Inconsistency	Indirectness	Publication bias	Other considerations	Quality	Ref.
Vaccination or contact with cases										
Having a sibling with no basic immunization	1	Observational	Not serious	Serious	Not serious	Not serious	Not serious		Very low	[31]
Incomplete vaccination (<3 doses)	18	Observational	Not serious	Not serious	Not serious	Not serious	Not serious	Large effect	Moderate	[[27], [28], [29], [30],[32], [33], [34], [35], [36], [37], [38],^41^,[43], [44], [45], [46], [47], [48]]
No booster vaccination in last five years	4	Observational	Not serious	Not serious	Not serious	Not serious	Not serious		Very low	[29,34,39,47]
No DTP dose in last ten years	1	Observational	Not serious	Serious	Not serious	Not serious	Not serious		Very low	[44]
Having contact with a diphtheria case	5	Observational	Not serious	Not serious	Not serious	Not serious	Not serious		Low	[[33], [34], [35], [36], [37]]
Having contact with a person with skin lesions	3	Observational	Not serious	Not serious	Not serious	Not serious	Not serious		Low	[33,34,36]

Underlying conditions										
Having a history of a chronic condition	2	Observational	Not serious	Serious	Serious	Not serious	Not serious		Very low	[34,36]
Having a history of eczema	1	Observational	Not serious	Serious	Not serious	Not serious	Not serious		Very low	[36]
Having a recent sore throat	2	Observational	Not serious	Serious	Serious	Not serious	Not serious		Very low	[36,48]
Having adenectomy (i.e., surgical removal of all or part of a gland)	1	Observational	Not serious	Serious	Not serious	Not serious	Not serious		Very low	[34]
Having cough or rhinorrhea four weeks prior to diphtheria onset	1	Observational	Not serious	Serious	Not serious	Not serious	Not serious		Very low	[36]
Having fever with myalgia four weeks prior to diphtheria onset	1	Observational	Not serious	Serious	Not serious	Not serious	Not serious		Very low	[36]
Having skin lesions or rash four weeks prior to diphtheria onset	1	Observational	Not serious	Serious	Not serious	Not serious	Not serious		Very low	[36]
Having tonsils	2	Observational	Not serious	Not serious	Serious	Not serious	Not serious		Very low	[36,48]
Malnutrition	2	Observational	Not serious	Not serious	Not serious	Not serious	Not serious		Very low	[37,44]

Knowledge and behaviour										
Abusing alcohol	2	Observational	Not serious	Serious	Serious	Not serious	Not serious		Very low	[36,42]
Attending social gatherings	2	Observational	Not serious	Serious	Not serious	Not serious	Not serious		Very low	[36,47]
Belief that vaccines are ineffective	1	Observational	Not serious	Serious	Not serious	Not serious	Not serious		Very low	[26]
Consumption of cheese, ice cream, or raw milk	1	Observational	Not serious	Serious	Not serious	Serious	Not serious		Very low	[33]
Consumption of factory-made yoghurt	1	Observational	Not serious	Serious	Not serious	Serious	Not serious		Very low	[33]
Drinking directly from faucets or a common water container	1	Observational	Not serious	Serious	Not serious	Not serious	Not serious		Very low	[47]
Having a guest spending a night in the house in last two weeks	1	Observational	Not serious	Serious	Not serious	Not serious	Not serious		Very low	[36]
Having fears or worries about vaccines and their safety	2	Observational	Not serious	Serious	Not serious	Not serious	Not serious		Very low	[26,37]
Having low knowledge of diphtheria	3	Observational	Not serious	Not serious	Not serious	Not serious	Not serious		Low	[26,32,37]
Having no knowledge of diphtheria vaccine boosters	1	Observational	Not serious	Serious	Not serious	Not serious	Not serious		Very low	[26]
Household member sought medical care in last two weeks	1	Observational	Not serious	Serious	Not serious	Not serious	Not serious		Very low	[36]
Household member spent night outside home in last two weeks	1	Observational	Not serious	Serious	Not serious	Not serious	Not serious		Very low	[36]
Infrequent bathing	3	Observational	Not serious	Serious	Not serious	Not serious	Not serious		Very low	[34,36,44]
Kissing someone outside family in last two weeks	1	Observational	Not serious	Serious	Not serious	Not serious	Not serious		Very low	[36]
Obtaining water from wheeled carrier	1	Observational	Not serious	Serious	Not serious	Serious	Not serious		Very low	[33]
Infrequent hand washing	2	Observational	Not serious	Serious	Not serious	Not serious	Not serious		Very low	[34,44]
Sharing a bed or bedroom	4	Observational	Not serious	Not serious	Serious	Not serious	Not serious		Very low	[35,36,38,44]
Sharing body and/or hand towels	2	Observational	Not serious	Serious	Not serious	Not serious	Not serious		Very low	[35,36]
Sharing cigarettes	1	Observational	Not serious	Serious	Not serious	Not serious	Not serious		Very low	[47]
Sharing toothbrushes	1	Observational	Not serious	Serious	Not serious	Not serious	Not serious		Very low	[34]
Sharing utensils, cups, glasses	4	Observational	Not serious	Not serious	Serious	Not serious	Not serious		Very low	[[34], [35], [36],^47^]
Smoking cigarettes	1	Observational	Not serious	Serious	Not serious	Not serious	Not serious		Very low	[36]
Staying in a dormitory	1	Observational	Not serious	Serious	Not serious	Not serious	Not serious		Very low	[44]
Talking or shaking hands with people at work	1	Observational	Not serious	Serious	Not serious	Not serious	Not serious		Very low	[36]
Travel history to area with diphtheria	2	Observational	Not serious	Not serious	Not serious	Not serious	Not serious		Very low	[35,37]
Wearing someone else's clothes before washing	1	Observational	Not serious	Serious	Not serious	Not serious	Not serious		Very low	[36]
Wearing the same clothes for multiple days prior to washing	1	Observational	Not serious	Serious	Not serious	Not serious	Not serious		Very low	[36]
Working in a dusty, noisy or smoky environment	1	Observational	Not serious	Serious	Not serious	Not serious	Not serious		Very low	[36]

Socioeconomic status										
Area of residence (urban or rural)	1	Observational	Not serious	Serious	Not serious	Not serious	Not serious		Very low	[36]
Attending school or kindergarten	2	Observational	Not serious	Serious	Not serious	Not serious	Not serious		Very low	[34,44]
Being a refugee	1	Observational	Not serious	Serious	Not serious	Not serious	Not serious		Very low	[36]
Being part of a large family or household	5	Observational	Not serious	Not serious	Not serious	Not serious	Not serious		Low	[26,32,35,36,40]
Being unemployed	2	Observational	Not serious	Serious	Not serious	Not serious	Not serious		Very low	[26,36]
Having a caregiver with low or no education	1	Observational	Not serious	Serious	Not serious	Not serious	Not serious		Very low	[26]
Having a father with low education	2	Observational	Not serious	Serious	Not serious	Not serious	Not serious		Very low	[32,35]
Having a low income	2	Observational	Not serious	Serious	Not serious	Serious	Not serious		Very low	[26,40]
Having a mother with low education	3	Observational	Not serious	Serious	Serious	Not serious	Not serious		Very low	[32,35,36]
Having an unemployed caregiver	2	Observational	Not serious	Serious	Not serious	Not serious	Not serious		Very low	[26,35]
Having contact with farm or pet animals in last two weeks	2	Observational	Not serious	Serious	Not serious	Not serious	Not serious		Very low	[36,44]
Having low or no education	2	Observational	Not serious	Serious	Not serious	Not serious	Not serious		Very low	[26,36]
Having no access to clean water at home	1	Observational	Not serious	Serious	Not serious	Not serious	Not serious		Very low	[32]
Having no standard toilet and washing facilities at home	1	Observational	Not serious	Serious	Not serious	Not serious	Not serious		Very low	[32]
Having poor ventilation at home	2	Observational	Not serious	Serious	Not serious	Not serious	Not serious		Very low	[32,37]
Having several siblings	1	Observational	Not serious	Serious	Not serious	Not serious	Not serious		Very low	[38]
Living in a house with few rooms	4	Observational	Not serious	Not serious	Not serious	Not serious	Not serious		Low	[26,[34], [35], [36]]
Living in a house with several children	2	Observational	Not serious	Serious	Not serious	Not serious	Not serious		Very low	[36,38]
Living in an overcrowded house	4	Observational	Not serious	Serious	Not serious	Serious	Not serious		Very low	[32,35,37,40]
Residential status (permanent or temporary)	1	Observational	Not serious	Serious	Not serious	Not serious	Not serious		Very low	[34]
Size of the household	3	Observational	Not serious	Not serious	Not serious	Not serious	Not serious		Very low	[32,40,44]
Type of home floor	1	Observational	Not serious	Serious	Not serious	Not serious	Not serious		Very low	[37]
Type of home wall	1	Observational	Not serious	Serious	Not serious	Not serious	Not serious		Very low	[37]
Type of house	3	Observational	Not serious	Serious	Not serious	Not serious	Not serious		Very low	[[34], [35], [36]]

Population-level factors										
Adult morbidity	1	Observational	Serious	Serious	Not serious	Not serious	Not serious		Very low	[53]
Conflict in past year	1	Observational	Serious	Serious	Not serious	Not serious	Not serious		Very low	[50]
Degree of urbanization	2	Observational	Serious	Serious	Serious	Not serious	Not serious		Very low	[51,53]
Diphtheria tetanus (DT) vaccine stockout	1	Observational	Serious	Serious	Not serious	Not serious	Not serious		Very low	[51]
DTP vaccine stockout	1	Observational	Serious	Serious	Not serious	Not serious	Not serious		Very low	[51]
Female literacy	1	Observational	Serious	Serious	Not serious	Not serious	Not serious		Very low	[51]
Health facility density	1	Observational	Serious	Serious	Not serious	Not serious	Not serious		Very low	[51]
Level of education	3	Observational	Serious	Not serious	Serious	Not serious	Not serious		Very low	[[52], [53], [54]]
Level of emissions into the air from stationary or mobile sources	1	Observational	Serious	Serious	Not serious	Not serious	Not serious		Very low	[53]
Level of sulfur dioxide in the air	1	Observational	Serious	Serious	Not serious	Not serious	Not serious		Very low	[53]
Levels of carbon monoxide, dust, or nitrogen dioxide in the air	1	Observational	Serious	Serious	Not serious	Not serious	Not serious		Very low	[53]
Median income	1	Observational	Serious	Serious	Not serious	Not serious	Not serious		Very low	[54]
Migration balance	1	Observational	Serious	Serious	Not serious	Not serious	Not serious		Very low	[53]
Morbidity in the rural population	1	Observational	Serious	Serious	Not serious	Not serious	Not serious		Very low	[53]
Morbidity in the urban population	1	Observational	Serious	Serious	Not serious	Not serious	Not serious		Very low	[53]
Mortality in the overall population	1	Observational	Serious	Serious	Not serious	Not serious	Not serious		Very low	[53]
Number of immunized children in areas with past conflict	1	Observational	Serious	Serious	Not serious	Not serious	Not serious		Very low	[50]
Number of immunized children in areas with ongoing conflict	1	Observational	Serious	Serious	Not serious	Not serious	Not serious		Very low	[50]
Ongoing conflict	1	Observational	Serious	Serious	Not serious	Not serious	Not serious		Very low	[50]
Percentage of households with no toilet facility	1	Observational	Serious	Serious	Not serious	Not serious	Not serious		Very low	[51]
Percentage of people below poverty line	1	Observational	Serious	Serious	Not serious	Not serious	Not serious		Very low	[53]
Percentage of overcrowded housing units	1	Observational	Serious	Serious	Not serious	Not serious	Not serious		Very low	[54]
Population expenditure per capita	1	Observational	Serious	Serious	Not serious	Not serious	Not serious		Very low	[52]
Population growth rate	1	Observational	Serious	Serious	Not serious	Not serious	Not serious		Very low	[53]
Population density	4	Observational	Serious	Not serious	Not serious	Not serious	Not serious		Very low	[[51], [52], [53], [54]]
School density	1	Observational	Serious	Serious	Not serious	Not serious	Not serious		Very low	[51]
Tuberculosis incidence	1	Observational	Serious	Serious	Not serious	Not serious	Not serious		Very low	[49]
Vaccination coverage	4	Observational	Serious	Not serious	Not serious	Not serious	Not serious		Very low	[[50], [51], [52], [53]]

**Table:** 

	Univariate		Multivariate	
Variables	**cOR**	**95% CI**	**aOR**	**95% CI**
Education	1.45	0.54–3.90		
Education of caretaker	1.07	0.52–2.15		
Occupation	1	0.48–2.05		
Occupation of caretaker	0.51	0.21–1.20		
Eligible for white ration card	0.68	0.33–1.37		
Mean persons sleeping in house	0.68	0.31–1.47		
Mean rooms in house	0.87	0.42–1.80		
Heard of diphtheria	3.73	1.69–8.17	3.56	1.58–8.04
*Knowledge about booster doses of diphtheria*				
Additional (booster) doses of diphtheria vaccine	0.22	0.10–0.51		
Booster doses are needed to stay protected	0.21	0.09–0.48		
How many booster doses are recommended	0.10	0.01–0.79		
What age a child should get the final booster dose	0.12	0.03–0.42		
*Attitude towards vaccines*				
Vaccines can prevent people from getting diseases	4.61	1.44–14.8	3.99	1.18–13.45
Vaccines are safe	1.34	0.29–6.32		
Fear or worries about vaccines	1.93	0.61–6.13		

**Table:** 

Variables	Cases	Controls	Cross-tabulated OR (95% CI)
*Vaccine doses*			
<3	61	171	4.57 (3.27–6.38)
≥3	156	1997	

**Table:** 

Variables	Cases	Controls	Cross-tabulated OR (95% CI)
*Vaccine doses*			
<3	41	18	2.92 (1.56–5.45)
≥3	82	105	

**Table:** 

Variables	Cases	Controls	OR (95% CI)
*Vaccine doses*			
<3	34	84	2.67 (0.96–7.42) ^#^
≥3	5	33	
*Years since last dose*			
≥5	6	2	12.7 (1.5–106.6)
0–4	19	50	

**Table:** 

Variables	Carriers	Controls	Cross-tabulated OR (95% CI)
*Social class*			
V	4	40	0.71 (0.21–2.41)
IV	10	71	
*Overcrowding*			
Present	3	53	0.31 (0.08–1.19)
Absent	11	61	
*Members in the family*			
≥7	5	57	0.56 (0.18–1.76)
<7	9	57	

**Table:** 

Variables	Cases	Controls	Cross-tabulated OR (95% CI)
*Vaccine doses*			
<3	87	29	8.37 (5.31–13.18) ^**#**^
≥3	175	488	

**Table:** 

Variables	Cases	Controls	Cross-tabulated OR (95% CI)
*Vaccine doses*			
<3	89	59	4.07 (1.84–9.03)
≥3	10	27	

**Table:** 

	Univariate		Multivariate	
Variables	**cOR**	**95% CI**	**aOR**	**95% CI**
Paternal education	5.50	1.560–19.392	5.5	1.560–19.392
Maternal education	3.077	0.862–10.978	0.921	0.169–5.009
Knowledge about diphtheria	3.836	1.081–13.604	2.434	0.647–9.159
Immunization: complete basic	1.517	0.649–3.547		
District	1.563	0.328–7.438		
Living in “pesantren”	0.481	0.058–4.018		
No. of people living in house (>2 vs <2 people)	1.511	0.607–3.763		
Ventilation	2.669	0.582–12.239		
Clean water source	2.0	0.826–4.844	1.161	0.448–3.007
Toilet and washing facilities	1.217	0.826–4.844		

**Table:** 

	*n* = 46		Univariate		Multivariate	
Variables	**Cases (%)**	**Controls (%)**	**RR**	**95% CI**	**RR**	**p value**
*Vaccine doses*						
<3	89	67	3.97 #	1.30–12.09		
≥3	11	33				
Contact with a diphtheria case	43	6	31.65	4.22–237.5	71.04	0.002
Contact with skin disease	22	7	3.84	1.16–12.7	41.91	0.04
Obtaining drinking-water from wheeled carrier	26	10	10.28	1.24–85.21	28.42	0.008
*Milk and milk products*						
Raw milk	17	7	4.25	0.83–21.9		
Factory-made yoghurt	60	24	3.88	1.71–8.8	14.92	0.003
Cheese	85	71	2.08	0.69–6.25		
Ice-cream	51	36	2.15	0.97–4.74		

RR, relative risk; CI, confidence interval; N/A, not available.

# Calculated using 2 × 2 cross-tabulation.

**Table:** 

Variables	Cases	Controls	Cross-tabulated OR (95% CI)	
*Vaccine doses*				
<3	10	26	1.61 (0.74–3.47)	
≥3	78	326		
	**Univariate**		**Multivariate**	
	**cOR**	**95% CI**	**aOR**	**95% CI**
*Bathing frequency*				
Once a day or less	1.73	1.05–2.86	1.74	1.04–2.91
Twice a day or more	1.00			

**Table:** 

	Univariate		Multivariate	
Variables	**cOR**	**95% CI**	**aOR**	**95% CI**
*Paternal factors*				
Low education level	0.8	0.4–1.4	0.6	0.3–1.5
Not working	1.3	0.6–2.8	1.1	0.5–2.5
*Maternal factors*				
Low education level	0.9	0.4–1.9	0.9	0.4–2.5
Not working	1.0	0.4–2.5	0.9	0.3–2.7
*Household factors*				
Type of house (apartment)	1.1	0.6–1.9	0.9	0.4–1.8
Family size of at least seven people (median)	0.7	0.4–1.3	0.6	0.3–1.2
Three or more rooms in house (mean)	0.9	0.5–1.6	0.8	0.4–1.7
Shared a bedroom with at least two people (mean)	2.1	1.0–4.2	2.8	1.2–6.6
Shared utensils and cups	1.2	0.4–3.2	1.1	0.3–3.3
Shared towels	1.3	0.7–2.5	0.9	0.4–1.9
*Immunization status*				
Non-vaccinated	2.3	1.2–4.6	2.6	1.2–6.0
*Source of infection*				
Travel history to area with diphtheria	1.2	0.4–3.2	0.5	0.2–1.7
Contact with a diphtheria case	9.1	2.6–31.5	10.6	2.6–43.6

**Table:** 

Variables	Matched OR	95% CI
Not vaccinated	19.23	4.79–77.17
*Exposure to infected person*		
Household member with diphtheria	7.46	1.46–37.99
Exposed to others with skin lesions	5.86	1.92–17.84
*Host factors*		
Person with tonsils	4.41	2.03–9.56
Fever and myalgia 4 weeks prior to reference date	2.67	1.29–5.52
History of chronic health problem	2.12	1.20–3.76
History of eczema	3.41	1.17–9.92
Bathed less than once a week	2.60	1.29–5.23
Wore someone else's clothes before washing them	0.7	0.5–1.1
Wore clothes >3 days before washing	1.1	0.7–1.6
Shared body and hand towel with family members	1.1	0.7–1.8
Shared hand towel at work	1.6	0.5–5.4
Shared hand towel with family members	1.2	0.8–1.8
Shared utensils outside family	1.3	0.4–4.3
Kissed someone outside of family in last 2 weeks	1.0	0.6–1.7
Kissed someone outside of family on lips in last 2 weeks	1.3	0.8–2.3
Shook hands on daily basis with the public at work	1.0	0.3–2.9
Talked or shook hands with 110 coworkers (contact within <1 m)	0.6	0.2–1.7
Nonsmokers	1.61	1.00–2.63
Heavy drinker (>14 drinks/week)	0.7	0.3–1.7
Being a refugee	0.9	0.2–2.7
*Exposure to animals*		
Touched farm animals in last 2 weeks	0.7	0.5–1.1
Milked a cow or goat in last 2 weeks	0.9	0.5–1.3
Pets living in home	1.0	0.7–1.4
*Household characteristics*		
Shared utensils, cups, or glasses with family	2.72	1.58–4.68
Shared a bed (with ≥1 person)	1.96	1.13–3.40
Apartment vs. house	0.7	0.4–1.1
No. of rooms in home (1–4 vs. 14)	1.7	1.0–2.8
No. of persons in household (>5)	1.0	0.7–1.4
Guest spent night in household 2 weeks prior to diphtheria onset	1.3	0.96–1.94
*Work environment*		
Dusty	1.8	0.5–5.4
Very noisy	1.4	0.5–3.6
Heavy cigarette smoke	1.9	0.7–5.2
*Exposure outside home 2 weeks before reference date*		
Attended church	0.6	0.3–1.2
Attended sports club	0.4	0.1–1.2
Attended social clubs	0.2	0.07–1.2
Attended movie	0.9	0.5–1.6
Attended funeral	0.7	0.5–1.2
Attended celebration	0.9	0.6–1.3
Cough, rhinorrhea, or sore throat 4 weeks before reference date	1.3	0.94–1.8

**Table:** 

	Bivariate		Multivariate	
Variables	**cOR**	**95% CI**	**aOR**	**95% CI**
DTP immunization status (i.e., having received three vaccine doses)	0.343	0.073–1.1.617		
Nutritional status	4.457	1.11–17.89	0.810	0.065–10.073
Mobility (i.e., travel to an area with diphtheria cases)	6.800	2.253–31.645	8.456	5.643–12.672
Source of contamination (i.e., contact with diphtheria case)	0.167	0.039–0.711	0.134	0.012–1.519
Knowledge of parent	1.111	0.306–4.037		
Attitude towards immunization program	0.889	0.244–3.243		
Home ventilation	1.615	0.370–7.049		
Home density of occupancy	0.833	0.203–3.427		
Home wall type	1.143	0.307–4.254		
Home floor type	1.167	0.302–4.512		

**Table:** 

Variables	Cases	Controls	OR (95% CI)
*Mother's occupation*			
Farmer	35	77	0.00 (0.00–**∞** i.e., infinity)
Non-farmer	3	2	
*Number of children <* *5 years in household*			
>2	26	60	1.43 (0.55–3.73)
≤2	11	19	
*Number of persons in household sharing bed*			
>4	23	46	0.39 (0.15–1.07)
≤4	15	33	
*Travel within 2 weeks*			
Yes	2	2	2.72 (0.23–32.78)
No	36	76	
*Chronic skin condition / lesion*			
Yes	0	0	N/A
No	37	79	
*Vaccine doses*			
<3	30	57	2.11 (0.72–6.17) ^#^
≥3	5	20	

**Table:** 

	Univariate		Multivariate	
Variables	**cOR**	**95% CI**	**aOR**	**95% CI**
*Number of doses*				
4	2.8	1.2–6.5	1.7	0.5–5.0
5	1.0^§^		1.0^§^	
	**Cases**	**Controls**		**Cross-tabulated OR (95% CI)**
*Years since last dose*				
≥5	11	37		1.70 (0.81–3.57) ^#^
<5	47	269		

**Table:** 

Variables	Carriers	Controls	Cross-tabulated OR (95% CI)
*Vaccine doses*			
<3	10	68	2.51 (1.07–6.31)
≥3	10	171	

**Table:** 

Variables	Cases / Carriers	Controls	Cross-tabulated OR (95% CI)
*Alcohol abuse*			
Yes	237	935	48.82 (27.20–87.61)
No	12	2311	

**Table:** 

Variables	Carriers	Controls	Cross-tabulated OR (95% CI)
*Vaccine doses*			
<3	1	205	0.56 (0.07–4.64)
≥3	6	683	

**Table:** 

Variables	Carriers	Controls	p value	Cross-tabulated OR (95% CI)
*DTP1 ≤* *10 years*				
0 doses	5	107	>0.99	
≥1 dose	16	336		
*DTP3 ≤* *10 years*				
0 doses	5	111	>0.99	0.94 (0.34–2.61)
≥1 dose	16	332		
*School*				
Not attended	17	906	0.12	
Attended	6	283		
*Dormitory*				
Not staying	23	1035	0.77	
Staying	4	154		
*Sharing a bed*				
Yes	4	143	0.67	1.26 (0.43–3.70)
No	23	1037		
*Household size, no. persons*				
≤4	13	585	>0.99	
>4	14	604		
*Bathing, times/day*				
<1	0	72	0.40	
≥1	27	1117		
*Handwashing, times/day*				
<3	4	247	0.11	
≥3	18	445		
*Livestock or pet animal*				
No	24	866	0.08	
Yes	3	323		

**Table:** 

Variables	Cases / Carriers	Controls	Cross-tabulated OR (95% CI)
*Vaccine doses*			
<3	44	305	1.12 (0.76–1.64)
≥3	87	673	

**Table:** 

Variables	Cases	Controls	Cross-tabulated OR (95% CI)
*Vaccine doses*			
<3	31	66	0.88 (0.52–1.46)
≥3	73	136	

**Table:** 

Variable	RR (95% CI)
Sharing cups	1.36 (1.06–1.74)

**Table:** 

Variables	Carriers	Controls	Cross-tabulated OR (95% CI)
*Vaccine doses*			
<3	22	505	2.07 (0.83–5.16)
≥3	6	285	
*Tonsillectomy*			
Yes	4	99	1.16 (0.40–3.42)
No	24	691	

**Table:** 

City	Tuberculosis coefficient	Standard error	p value	R^2^	N
Boston 1916–1923	0.14	0.07	0.047	0.51	411
Chicago 1916–1923	0.11	0.03	0.0004	0.66	409
Detroit 1915–1923	0.12	0.05	0.01	0.68	453
New York 1916–1923	0.03	0.04	0.41	0.63	408
New York w/o 1921	0.07	0.04	0.05	0.64	357
Philadelphia 1907–1923	0.06	0.03	0.04	0.57	823

**Table:** 

	Bivariate		Multivariate	
Variables	**cOR**	**95% CI**	**aOR**	**95% CI**
No. of immunized children	1.02	1.01–1.03	1.04	1.01–1.058
Conflict in past year	0.59	0.33–1.04	3.25	0.16–67.70
Ongoing conflict	1.89	1.20–2.99	11.21	1.29–97.69
No. of immunized children in areas with past conflict	0.99	0.99–1.00	0.98	0.95–1.02
No. of immunized children in areas with ongoing conflict	1.01	1.00–1.02	0.98	0.95–1.01

**Table:** 

Variables	Initial OLS (SE)	Final OLS (SE)	Final GWR
DTP3 coverage	0.177 (0.488)		
Health facility density	0.015 (0.007)⁎	0.015 (0.005)⁎⁎	0.015
Improved water source	0.003 (0.003)		
Female literacy	−0.026 (0.007)⁎⁎⁎	−0.024 (0.006)⁎⁎⁎	−0.024
No toilet facility	< −0.001 (0.004)		
School density	−0.001 (0.002)		
Population density	< −0.001 (<0.001)		
Urbanization	0.007 (0.003)⁎⁎	0.006 (0.002)⁎⁎	0.006

OLS, ordinary least squares; GWR, geographically weighted regression; SE, standard error.

⁎ *p* < 0.05.

⁎⁎ *p* < 0.01.

⁎⁎⁎ *p* < 0.001.

**Table:** 

Variables	r	p value
*Population density*		
2010	0.002	0.991
2011	0.072	0.668
*Education level*		
2010	0.059	0.725
2011	0.089	0.089
*DTP3 coverage*		
2010	0.424	0.008
2011	0.221	0.183
*DT coverage*		
2010	0.348	0.032
2011	0.198	0.232
*Healthy home*		
2010	0.125	0.454
2011	0.104	0.232
Per capita *expenditure*		
2010	0.037	0.823
2011	0.065	0.700

**Table:** 

Variables	Regression coefficient (B)	Standard error	χ^2^ walda	p value	95% PI for odds ratio
*Environmental factors*					
Sulfur dioxide	0.226	0.055	16.7	<0.001	1.125–1.396
*Social factors*					
Vaccination	−0.043	0.017	6.6	0.001	0.926–0.990
*Generalized environmental factors (indices of human development)*					
Living conditions, incl. Population density	0.037	0.009	18.0	<0.001	1.020–1.056
*Expeditors of risk*					
Morbidity rate of city residents	0.153	0.063	5.9	0.001	1.031–1.318

**Table:** 

Variables	Regression coefficient (B)	p value	F
Percentage of people below poverty	0.023	<0.05	37.196
Population density	0.004	>0.05	0.002
Percentage living in overcrowded households	−0.092	>0.05	0.590
Median education	0.162	>0.05	1.046
Median income	0.074	>0.05	0.260

**Figure:**
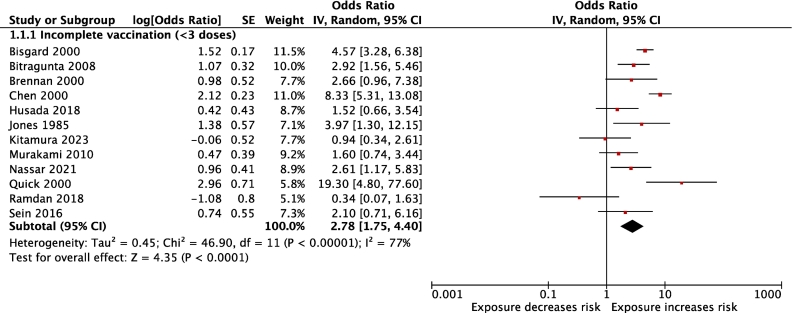


**Figure:**
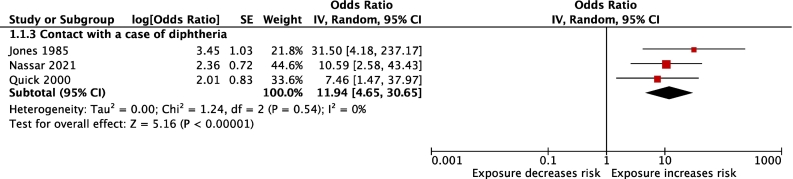


## Appendix 2 Search strategy

**EMBASE and MEDLINE**

**PubMed and Web of Science**

## Appendix 3 Newcastle-Ottawa Scale assessment scores

**Case-control studies**

**Cohort study**

**Cross-sectional studies**

## Appendix 5 Reported risk estimates

The tables below present all the risk estimates from each included study.

**Case-control studies**

Allam 2016.

aOR, adjusted odds ratio; CI, confidence interval; cOR, crude odds ratio.

Bisgard 2000.

OR, odds ratio; CI, confidence interval; N/A, not available.

Bitragunta 2008.

OR, odds ratio; CI, confidence interval; N/A, not available.

Brennan 2000.

OR, odds ratio; CI, confidence interval; N/A, not available. ^#^ Calculated using 2 × 2 cross-tabulation.

Chandra 1973.

OR, odds ratio; CI, confidence interval; N/A, not available.

Chen 2000.

OR, odds ratio; CI, confidence interval; N/A, not available.

Faria 1971.

OR, odds ratio; CI, confidence interval.

Husada 2018.

aOR, adjusted odds ratio; CI, confidence interval; cOR, crude odds ratio.

Jones 1985.

Murakami 2010.

aOR, adjusted odds ratio; CI, confidence interval; cOR, crude odds ratio.

Nassar 2021.

aOR, adjusted odds ratio; CI, confidence interval; cOR, crude odds ratio.

Quick 2000.

OR, odds ratio; CI, confidence interval.

Ramdan 2018.

aOR, adjusted odds ratio; CI, confidence interval; cOR, crude odds ratio.

Sein 2016.

OR, odds ratio; CI, confidence interval; N/A, not available. ^#^ Calculated using 2 × 2 cross-tabulation.

Vitek 1999.

aOR, adjusted odds ratio; CI, confidence interval; cOR, crude odds ratio. ^§^ Reference group.

**Cross-sectional studies**

Belsey 1969.

OR, odds ratio; CI, confidence interval.

Harnisch 1989.

OR, odds ratio; CI, confidence interval.

Kalapothaki 1984.

OR, odds ratio; CI, confidence interval.

Kitamura 2023.

OR, odds ratio; CI, confidence interval.

Marcuse 1973.

OR, odds ratio; CI, confidence interval.

Miller 1972.

OR, odds ratio; CI, confidence interval.

Ohuabunwo 2005.

OR, odds ratio; CI, confidence interval.

Trichopoulos 1972.

OR, odds ratio; CI, confidence interval.

**Ecological studies.**

Coleman 2018.

R^2^ coefficient of determination.

Dureab 2018.

aOR, adjusted odds ratio; CI, confidence interval; cOR, crude odds ratio.

Ikejezie 2022.

Izza 2015.

r, Spearman's rank correlation coefficient.

Podavalenko 2018.

PI, prediction interval.

Quesada 1979.

PI, prediction interval.

## Appendix 6 Sensitivity analyses

We conducted a sensitivity analysis after removing studies that did not adjust for potential confounders. The analysis was conducted only for incomplete vaccination (i.e., having received less than three primary doses of the diphtheria vaccine). The results of the sensitivity analysis (POR = 2.8, 95% CI = 1.8–4.4) were concordant with the results of the main meta-analysis, in terms of both magnitude and direction of effect.

**Sensitivity analysis of incomplete vaccination after removing studies that did not adjust for potential confounders.**

We conducted a sensitivity analysis after removing a study that had outlier values for several risk factors. The results of the sensitivity analysis (POR = 11.9, 95% CI = 4.7–30.7) differ from those of the main meta-analysis, in terms of both magnitude and direction of effect.

**Sensitivity analysis of contact with a diphtheria case after removing a study that had outlier values for several risk factors.**
